# Evaluating effects of meal delivery on the ability of homebound older adults to remain in the community via a pragmatic, two-arm, randomized comparative effectiveness trial: study protocol for the Deliver-EE trial

**DOI:** 10.1186/s13063-024-08635-3

**Published:** 2024-11-22

**Authors:** Kali S. Thomas, Kimberly P. Bernard, Melissa Clark, Laura Dionne, Alison Fisher, Emily Gadbois, Jill Harrison, Lisa Juckett, Julie Locher, Patricia Risica, Tamara Sequeira, Lucy Theilheimer, Roee Gutman

**Affiliations:** 1 Johns Hopkins School of Nursing, 525 North Wolfe Street, Baltimore, MD 21205 USA; 2 Center for Gerontology & Healthcare Research, Brown University, Providence, RI USA; 3 Department of Health Services, Policy and Practice, Brown University, Providence, RI USA; 4 School of Public Health, Brown University, Providence, RI USA; 5 School of Health and Rehabilitation Sciences, The Ohio State University, Columbus, OH USA; 6 Heersink School of Medicine, University of Alabama at Birmingham, Birmingham, AL USA; 7 Department of Behavioral and Social Sciences, Brown University, Providence, RI USA; 8 Meals On Wheels America, Arlington, VA USA; 9 Department of Biostatistics, Brown University, Providence, RI USA

**Keywords:** Food insecurity, Healthcare costs, Meal delivery, Older adults, Community independence, Aging in place, Healthcare utilization, Quality of life

## Abstract

**Background:**

As food insecurity and healthcare costs are linked, healthcare entities (i.e., providers, healthcare systems, insurers) are increasingly interested in identifying and providing solutions to address food insecurity among their patients. Home-delivered meals are one long-standing solution to address food insecurity among homebound older adults. However, there is limited evidence about what mode of delivery is most effective in promoting community independence, reducing healthcare utilization, and improving quality of life as well as how these outcomes may vary as a function of people’s preferences for how meals are delivered to them.

**Methods:**

With extensive stakeholder input, we designed and implemented a pragmatic randomized comparative effectiveness study in which we will enroll 2300 older adults on waiting lists at home-delivered meals programs across the country and randomize them to receive for 6 months, either (1) weekday lunchtime meals delivered by a local volunteer or driver who also provides socialization and wellness checks or (2) biweekly delivery of 10 frozen meals to participants’ homes. Participant data will be combined with Centers for Medicare and Medicaid Services (CMS) data to calculate post-randomization institutional vs. community days. Baseline and 3-month surveys will evaluate secondary outcomes (e.g., food insecurity, loneliness, quality of life) and exploratory outcomes (e.g., nutritional risk). To examine heterogeneity of treatment effects, we will test for interactions between the two types of meal delivery and participants’ preferred mode of meal delivery as well as participants’ living arrangements.

**Discussion:**

This research will be the first to prospectively evaluate the comparative effectiveness of the two predominant meal delivery options. The knowledge generated from this research will be of value to healthcare providers, health systems, payers, community-based organizations, older adults, and their families, because it will identify the mode of meal delivery that best meets homebound older adults’ needs and promotes community independence. In addition, the experience of working closely with stakeholders in designing and conducting this trial will be useful to researchers seeking to engage with stakeholders in the development and evaluation of complex social service interventions while balancing regulatory, resource, and human subjects research considerations.

**Trial registration:**

ClinicalTrials.gov. NCT05357261. Registered on May 02, 2022

**Supplementary Information:**

The online version contains supplementary material available at 10.1186/s13063-024-08635-3.

## Introduction

Food insecurity among older adults is a critical public health issue with implications for families, the healthcare system, and society. Food insecurity disproportionately affects older adults who are racial or ethnic minorities, those with lower incomes, those who are disabled, and those living in non-metropolitan areas [1–3]. Food is a basic human need; among many food-insecure older adults, the “need for food competes with the need for other basic necessities such as medication, housing, utilities, and transportation” [4]. While food-insecure older adults can sometimes afford their food needs, they may not have the resources to access or prepare food because of lack of transportation, functional limitations, or health problems [1, 5].

Among older adults, food insecurity is associated with poor health status and health outcomes, and accounts for an estimated $130 billion annually in healthcare expenses[1, 6, 7]. Food insecure older adults are twice as likely to report fair or poor health, 50% more likely to have diabetes, three times more likely to suffer from depression, 30% more likely to have functional impairment, and nearly 60% more likely to have congestive heart failure or experience a heart attack [7]. Food insecurity can also pose challenges to chronic disease management and is associated with higher healthcare utilization and costs [8, 9]. Recognizing the link between food insecurity and health, health care entities (i.e., providers, healthcare systems, insurers) are increasingly interested in addressing food insecurity, among other social risk factors, through non-medical services such as home-delivered meals for older patients [10–15]. For example, in 2020, 42 states covered home-delivered meals in their Medicaid program [16]; and in 2022, almost 2 million Medicare beneficiaries were enrolled in a Medicare Advantage plan that offered a meals benefit [17].

There is limited evidence about which kind of home-delivered meals program is best for older adults. Home-delivered meals, long provided through a network of community-based organizations funded through the Older Americans Act, state dollars, and charitable contributions, grew out of a need to promote food security, socialization, and independence among older adults [18]. Until recently, meals have most commonly been delivered to older adults’ homes at lunchtime on weekdays using a volunteer or paid driver. Drivers often socialize and develop relationships with their clients, including providing an informal wellness check, sharing concerns about the client with the program, and often provide assistance beyond delivering meals (e.g., bringing in the mail, moving tripping hazards, troubleshooting air conditioning problems) [19]. It is believed that these social interactions contribute to the positive impact home-delivered meals have had on the health and healthcare utilization of a highly vulnerable population of homebound older adults experiencing food insecurity [20–25]. In recent years, vendors who provide mailed, frozen meals have gained popularity because they can provide a lower cost alternative: weeks’ worth of meals mailed to participants in one bulk package. While these meals have the same nutritional standards as daily-delivered meals (1⁄3 of older adults’ Dietary Reference Intake) [26], there is limited evidence on how satisfaction with, and the health benefits of, the frozen, mailed meals may differ from the traditional daily-delivered meals provided by community-based organizations. Prior research suggests that older adults who receive daily-delivered meals may have lower rates of loneliness and falls than those who receive frozen, once-weekly delivered meals from these community-based organizations [24, 25]; however, overall differences in health and healthcare utilization attributable to the two meal delivery methods remain unknown.

An important aspect of addressing food insecurity among homebound older adults is doing so in a way that aligns with their preferences. However, there is limited evidence about which mode of home-delivered meals older adults prefer. Many healthcare entities and community-based organizations strive for person-centered care, but there is little evidence to guide them on what mode of meal delivery older adults prefer or how receiving meals that older adults prefer may affect satisfaction and ultimately outcomes. Therefore, decision-makers have little evidence on either (a) which mode of delivery is more effective in reducing costly healthcare utilization and improving older adults’ quality of life and (b) how the effect of meal delivery on health and utilization outcomes may vary as a function of older adults’ meal delivery preferences.

To address these gaps in knowledge, we are conducting a multi-site, randomized pragmatic comparative effectiveness study with two parallel arms (the two modes of meal delivery) and a primary endpoint of institutional healthcare use in the 6 months after enrollment. The objective of this paper is to describe our protocol for the trial and present how it was shaped by stakeholder input (e.g., older adults receiving meals, meal providers, healthcare representatives). We specifically highlight the regulatory and resource challenges in responding to stakeholder input when designing and implementing this study.

## Methods

### Overview

The Deliver-EE: Evaluating Effects of Meal Delivery on the Ability of Homebound Older Adults to Remain in the Community Via A Pragmatic, Two-Arm, Randomized Comparative Effectiveness Trial, also known as The Deliver-EE Trial, was developed and is executed in partnership with Meals on Wheels America (MOWA), MOWA’s participating member programs, and a diverse group of stakeholders. In the conceptualization phase of the project, we worked with stakeholders to determine the most feasible study design and to anticipate system and local contextual barriers to study implementation, such as variation in meal prices by region. As a result of this engagement in the planning phase, we revised the analytic plan and site selection criteria and made changes to the protocol before submitting our application for funding consideration.

After funding was received (11/2021), we recruited 26 individuals to serve on two advisory panels: the Lived Experience Panel and the Systems’ Perspective Panel. Individuals serving on the Lived Experience Panel represent the frontlines of meal delivery and have unique knowledge about the interpersonal interactions and lived experiences of receiving and delivering meals. These members include older adults receiving meals from participating programs, care partners of older adults receiving meals, and volunteer and paid meal delivery drivers. The second panel represents the “Systems’ Perspective.” Members include representatives from the financial, clinical, regulatory, and operational environments in which meal delivery to food insecure older adults operates. These individuals include representation from healthcare plans, integrated care organizations, geriatric home care clinician researchers (including a practicing geriatrician with a home care practice and an advanced nurse practitioner who makes house calls), a registered dietitian, an expert in the integration of social services and medicine, and three home-delivered meals volunteer drivers from programs not participating in the trial. The panels began meeting with the research team in February 2022.

Brown University ceded regulatory oversight of the study to Advarra, Inc., an independent Institutional Review Board (IRB). This study was approved by the Advarra IRB as a minimal risk study. We do not have a Data and Safety Monitoring Board; any protocol deviations are reported to the Advarra IRB. Monthly enrollment reports are created by the research team and shared with the trial funder. Protocol amendments are shared with the funder and updated in the contract and clinical trial registry. In writing this report, we utilized the SPIRIT checklist to ensure the completeness of our trial protocol [27].

### Setting

The Deliver-EE Trial began enrollment in May 2022 and is expected to reach its enrollment target by December 2024. We are targeting enrollment of 2300 homebound older adults ages 66 years and older (to allow for a 1-year look back of their Medicare data) on waiting lists at ten Meals on Wheels programs across the country (a list of sites and their location is included in the Acknowledgements). Sites vary by geographic location, size, and demographic characteristics. We selected our study sites based on the size of their waiting lists and their capacity to serve participants. All participating programs agreed to provide administrative support, including sharing their waitlist and meal-delivery information, as well as provide meals using their standard processes to participants enrolled in the daily-delivered meals arm.

### Inclusion and exclusion criteria

To participate in the study, individuals must be 66 years of age or older, be on a waiting list at a participating program, and reside in a program’s daily service area (all areas served by programs may not have daily deliveries because of logistical and resource limitations). Individuals whose primary language is not English or Spanish, who have end stage renal disease requiring specialized diets, and those who are unable to participate in a phone survey/interview (e.g., difficulty hearing and speaking, lack of comprehension of study purpose, do not have a working phone) are ineligible for participation.

### Modes of meal delivery

The two treatment arms of the intervention are the two predominant modes of meals delivery: drop-shipped frozen meals and daily-delivered meals. All participants randomized to the daily-delivered meals arm receive lunch-time meals that meet national Title III requirements (i.e., (1⁄3 of older adults’ Dietary Reference Intake) [26]) delivered to their homes, five times per week, by a Meals on Wheels program employee/volunteer who socializes with the participant and provides an informal wellness check—all of which represent usual practices of Meals on Wheels programs nationwide and are tailored to the needs of participants and each program’s available resources. All participants randomized to the drop-shipped meals arm receive 10 frozen meals that are mailed to their home every 2 weeks from a vendor who provides meals meeting the same nutritional guidelines as the participating Meals on Wheels programs for 6 months. Participants may opt to stop receiving meals at any time and programs may discontinue meal service for noncompliance. Participants who discontinue meals will remain enrolled in the study and their data will be tracked. At the completion of the 6-month study period, participants continue to receive meals from participating programs pending meeting eligibility criteria and availability of resources.

### Recruitment and randomization

**Fig. 1:**
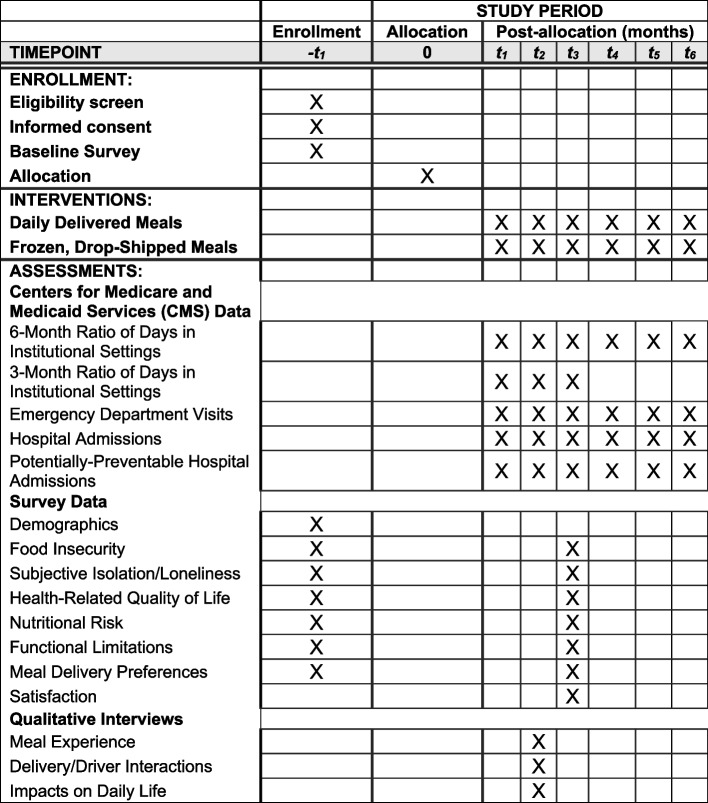
Deliver-EE SPIRIT study schedule

We developed the recruitment plan in partnership with participating programs and our advisory panels. To identify potential participants, Meals on Wheels program staff share demographic and contact information about individuals on their waiting lists who meet inclusion criteria with the research team. The research team sends an introductory packet about the study by mail and then contacts these individuals by telephone to screen, obtain informed consent, conduct the baseline survey, and randomize them to the two treatment arms (see Fig. 1).

Participants are assigned by the research team to receive either daily-delivered meals or drop-shipped meals with a 1:1 allocation using stratified randomization. Randomization stratification variables include the program, the personal meal preference (daily/no preference vs. frozen), and the living arrangement (alone vs. with others). Randomization occurs at the end of the baseline survey.

### Masking

Participants are unable to be blinded to their randomized condition. The research staff conducting the follow-up surveys and the biostatistician conducting the outcomes analyses are blinded to the randomized condition.

### Consent

The informed consent process begins with the potential participant receiving an informational packet containing details about the study. The packet contains four items: (1) a welcome letter that provides an overview of the research opportunity, (2) a Letter of Support from the participating program, (3) a “Frequently Asked Questions” document addressing potential questions in user-friendly language, and (4) an Informed Consent Document.

Approximately 2 weeks after the packet is mailed to the potential participant, a trained interviewer from the Brown University Survey Research Center calls the potential participant at their preferred telephone number provided by the program. If the potential participant is not reached in the first attempt, the interviewer makes a minimum of two additional telephone calls and one text message, if applicable, at different hours and days over approximately 2 weeks. When a potential participant is successfully contacted, the interviewer reviews the most salient points from the informed consent document using an IRB-approved script, answers any questions about the study, collects a verbal consent to participate, and immediately begins the baseline survey.

### Data collection

Survey data are being collected for all participants pre-intervention (baseline) and 3 months later (follow-up). Survey questions include items to assess demographics, preference for daily or drop-shipped meals, and health and well-being (e.g., food insecurity, health-related quality of life). To assess our exploratory outcome, nutritional risk (described below), we are conducting a longer survey that includes the Dietary Screening Tool with 600 randomly-selected participants. Additionally, we are conducting semi-structured telephone interviews with approximately 50 participants two months after they begin receiving services to collect information about their experiences receiving meals, how these services may have impacted their lives, and their satisfaction with these services. We are purposefully selecting participants for the semi-structured interviews across programs, treatment arm, preference, and living situation. A member of the research team contacts a participant selected for a qualitative interview and invites them to participate. These interviews take approximately 30–45 min to complete. Additional data sources include participant and service data from participating Meals on Wheels programs and claims, assessment, and enrollment files from the Centers for Medicare and Medicaid Services (CMS). We obtained a HIPAA waiver of authorization to use the CMS data. See appendix for information on data management and maintaining confidentiality.

### Outcomes and measures

When writing the research proposal for the funding that supports this work, we established the relevance of the proposed study with a broad group of stakeholders, concentrating our efforts on intended end users of the results: healthcare entities, meal providers, homebound older adults, their care partners, and the drivers who deliver meals. These stakeholder partners identified the person-centered outcomes (see Table 1) to include in the study.

**Table 1:** Deliver-EE clinical trial outcomes

Primary/secondary/exploratory	Name of outcome	Specific measure to be used	Timepoints	Data
Primary	6-month ratio of days in institutional settings	Ratio between the number of days that are spent in an institutional setting (i.e., acute care hospital, psychiatric hospital, long-term care hospital, nursing home, or skilled nursing facility) and the number of days alive in the 6 months following receipt of meals	6-month	Medicare claims
Secondary	3-month ratio of days in institutional settings	Defined similarly to the primary outcome, but assesses the immediate (3-month) impact of meals on days spent in institutional settings	3-month	Medicare claims
Secondary	Food insecurity	The 6-item Household Food Security Scale Short Form [27]	3-month	Survey
Secondary	Subjective isolation/loneliness	UCLA 3-Item Loneliness Scale [28]	3-month	Survey
Secondary	Health-related quality of life	CDC’s Health-Related Quality of Life 4-question core modules, called the “Healthy Days Measures” [29]	3-month	Survey
Exploratory	Nutritional risk	Dietary Screening Tool [30]	3-month	Survey
Exploratory	Emergency department visits	Number of emergency department visits	6-month	Medicare claims
Exploratory	Hospitalizations	Number of acute care hospital admissions	6-month	Medicare claims
Exploratory	Potentially preventable hospitalizations	Number of AHRQ Adult Ambulatory Care Sensitive Condition Admissions [57]	6-month	Medicare claims

The primary study outcome, prioritized by a variety of stakeholders and measured via CMS data for all enrolled participants, is the ratio of the number of days that are spent in an institutional setting (i.e., acute care hospital, psychiatric hospital, long-term care hospital, nursing home, skilled nursing facility) to the number of days alive in the six months following the start of meal delivery.

As a secondary outcome, we are also examining this ratio of days in institutional settings for a period of 3 months after the start of meal delivery. Other secondary outcomes include participant-reported food insecurity, loneliness, and health-related quality of life that are collected during the baseline and follow-up surveys. Food insecurity is measured with the Household Food Security Scale Short Form, which includes six questions about being able to afford food in the last 30 days [27]. Loneliness is measured with the UCLA 3-item loneliness scale, including frequency questions about lacking companionship, feeling left out, and feeling isolated [28]. Four questions are included from the CDC’s Health-Related Quality of Life module to assess quality of life [29]. We chose to assess our secondary person-centered outcomes halfway through the follow-up period because (a) we were able to detect an effect in subjective isolation/loneliness at three months in our prior pilot study [23] and (b) we wanted to minimize potential loss to follow-up for the secondary outcomes.

In response to feedback from our advisory panels, we include the number of emergency room visits, all cause hospitalizations, and potentially preventable hospitalizations ascertained from the CMS data as exploratory outcomes. Additionally, we assess nutritional risk using 26-item food frequency questions during the baseline and follow-up surveys using the Dietary Screening Tool (DST) [30] as an exploratory outcome.

Demographic measures that are included in the baseline survey are marital status, race, ethnicity, level of education, military service, receipt of Veterans Health Administration services, and number of other individuals living in the home. Data on gender and age are provided by the participating programs. Meal delivery data are also provided by the participating programs, including referral status (e.g., accepted or declined meals), meal delivery dates, the number of meals provided on each date, reasons for referral declines, and reasons for meal service terminations. Comorbidities are summarized from the CMS administrative data. Additional questions are included in the follow-up survey to assess participants’ ratings of the meal program overall, delivery method, ease of meal preparation, and taste and amount of food provided. Participants are also asked how much of the food they eat, whether they are still receiving meals, and, if not, why they are no longer receiving meals.

### Sample size

In a preliminary analysis of observational data, we observed that the proportion of days spent in an institution was 0.04 (SD = 0.12) for Meals on Wheels clients in the 6 months after receiving meals compared to 0.07 (SD = 0.19) among similar individuals that were not receiving meals. Because we are comparing two modes of meal delivery, we assume that the difference in proportions between the two modes of meal delivery is 0.02. Using a two-sample *t*-test, and assuming a 1:1 randomization, two-tailed test, and α = 0.05, we need a sample size of 1984 participants, 992 in each arm, to detect a 2% difference in the proportion of days spent in institutional settings to achieve 80% power. In the rare case we have difficulty deterministically linking participant data to their CMS data, we will use probabilistic linking techniques [22, 31]. Given that in prior research we have been able to identify 1-to-1 matches for 87% of Meals on Wheels clients using probabilistic methods, we are targeting enrollment of 2300 participants to ensure that we will have adequate sample size if we have unexpected linking challenges.

We have found during the study that some participants live in the same household with another participant. In situations where participants are living together, we are randomizing the entire household to the same meal assignment. We estimated the impacts on power for scenarios spanning from 10% of participants living in shared households to 30% using a sample size formula for cluster randomized trials [32]. With 1984 participants, only at the highest level of shared household proportions (30%) with highly correlated outcomes (0.1 ICC) did the power decrease from 0.80 to 0.79. We are monitoring the frequency of shared households and will adjust our sample size, as necessary.

### Statistical methods

#### Primary outcome analysis

Our primary analysis is an intention-to-treat analysis, meaning that we will assess outcomes for all participants assigned to each group regardless of how long they continue to receive meals. In randomized experiments, the distribution of all the covariates should be similar on average. However, because there are many covariates (e.g., age, education), one may expect that some will suffer from slight imbalances. Using the baseline survey data and participants’ prior Medicare claims, we will test for balance in these covariates between the two arms by comparing means and proportions. To address any minor imbalances and to obtain a more efficient estimate, we will use regression adjustments to estimate the marginal average treatment effect. Because outcomes of individuals within the same household may be correlated, we will use a hierarchical model to adjust for these correlations. Standard errors will be obtained using the bootstrap procedure.

#### Heterogeneity in treatment effects

We hypothesize that the treatment effect will differ among preference groups, with participants who receive a meal delivery method that is concordant with their preferences having better outcomes than those who do not receive a meal delivery method concordant with their preference. We will therefore conduct stratified analyses by each of the four preference groups: (1) preferred and received daily meals, (2) preferred daily meals and received drop-shipped meals, (3) preferred and received drop-shipped meals, (4) preferred drop-shipped meals and received daily meals. We will compare the ratio of days in institutional settings among the intervention groups and examine if they are significantly different across subgroups. To adjust for multiple comparisons, we will perform Bonferroni correction [33].

Based on research examining factors predicting institutionalization [34, 35], we expect that the treatment effect will differ between participants’ living alone from those living with others. We will conduct subgroup analyses to determine if differences in the intervention effect differs between the two living arrangement groups. We hypothesize that the difference in the primary and secondary outcomes between the two arms will be greater for participants who live alone than those who live with others. To test for the significance of heterogeneity of treatment effect by living arrangement, we will use the common *t*-distribution [36].

#### Supplementary analyses

Because we are interested in the effect of the intervention and not only the effect of being randomized to receive the intervention, we will conduct a supplementary compliance analysis [37, 38]. We will examine the effect of the intervention only among participants who received the meals for at least 4 weeks in both arms and for participants who received meals for at least 3 months (consistent with the timeline for our secondary outcome measures). To do so, we will use the principal stratification framework, which has been proposed as a plausible solution to address non-compliance in randomized trials [39]. We will use the Bayesian framework to identify compliers [37, 40]; this will allow us to examine the sensitivity of our results to commonly-used identifying assumptions (e.g., monotonicity, exclusion restriction). With our modeling assumptions, we can identify characteristics that are indicative of compliance with assigned intervention, which would allow for better tailoring of meals in the future.

We do not expect that the different interventions will affect mortality. However, we will monitor whether there is a differential difference in mortality, by arm. If one is observed, as a supplementary analysis, we will use principal stratification analysis to account for differential mortality [41]. We will also conduct exploratory analyses to determine if the treatment effects vary by comorbidities ascertained from CMS data, as well as by baseline food insecurity status and baseline loneliness/social isolation.

#### Secondary and exploratory outcomes analysis

For our secondary outcomes, we will examine claims data for the 3-month institutional care use measure and conduct pre- and post-intervention telephone surveys for the patient-reported secondary outcomes. Pre- and post- loneliness, food insecurity, and health-related quality of life measures will be directly compared. For our exploratory outcome, nutritional risk, we will directly compare pre- and post-Dietary Screening Tool scores for the sub-sample of 600 randomly selected participants. For the exploratory outcomes requested by our advisory panels (emergency room visits, hospitalizations, and avoidable hospitalizations), we will use CMS data and compare the rates and times to events between the two arms.

#### Addressing missing data

To address missing data for our secondary outcomes because of participant non-response to survey questions, we will assume that the variables are missing at random (MAR) and will utilize multiple imputation to generate 100 datasets in which all of the variables are fully observed [42]. We will analyze each of the 100 datasets separately, combining final results into point and interval estimates using common combination rules [42].

#### Qualitative data analysis

With participants’ consent, interviews will be recorded and transcribed for data analysis. Following each interview, we will write detailed summaries and interviewer perceptions of the interview. Qualitative transcripts will be analyzed using a modified grounded theory approach [43]. We will first develop a preliminary coding scheme based on the interview questions and informed by interview summaries, then adjust the scheme in an iterative process to add codes and refine code definitions. Additional codes will be applied to the text to account for new and unanticipated material from the interviews. The resulting coding scheme will thus reflect both the a priori codes and areas of interest from the interview protocols as well as any unexpected and new findings. As we continue this analytic process, we will record the potential trends and patterns we identify in participants’ responses in an ongoing “audit trail” [44]. The audit trail will serve as an analytic diary to record developing themes, ideas about the narrative data, and analytic decisions. The team will identify and discuss potential themes and will look both for prominence (or prevalence) of the themes across the narratives as well as search for competing interpretations. Interpretations and identified themes will be presented to the advisory panels to elicit feedback, alternative perspectives, and explanations [45].

The integration of the qualitative and quantitative data will be achieved through regular team meetings to discuss emerging findings. For example, the investigators leading the qualitative aspect of the study will provide regular updates and preliminary findings to the research team and advisory panels. Similarly, updates regarding the quantitative findings will be presented and appropriately enhanced by qualitative data, as needed. The groups will engage in discussions about the potential interpretations of the qualitative and quantitative findings as well as how these findings may relate to the outcomes that are observed.

### Stakeholder engagement

Our previous experience with stakeholder engagement in research reinforced our professional ethos, commitment, and attention to a growing body of literature suggesting that stakeholder engagement is critical to improving the relevancy and uptake of research findings [19, 46, 47]. Therefore, we sought out to meaningfully engage end users through the life course of the project, from conceptualization to evaluation [9, 48, 49]. The shared overall goals for stakeholder engagement in this study are to engage people with lived experience and systems-level knowledge who can holistically represent the interdependent elements of meal delivery to food insecure older adults to (1) inform our understanding of what matters most to older adults and systems, (2) provide strategies to overcome challenges in the conduct of the study, (3) enhance dissemination and uptake of study findings, and (4) identify opportunities for future research. Together, both stakeholder panels provide a holistic view of the inter-related coordination and integration that must occur for adoption of study results into policy and practice.

The protocol for engaging stakeholders in Deliver-EE, including recruitment, logistics, methods to orient stakeholders to the study, assessment of priorities for engagement, obtaining input and feedback on research activities, and evaluating engagement activities is published elsewhere [50]. In Table 2, we present examples of protocol changes we made in response to stakeholder feedback in the start-up phase of the project. In Table 3, we present examples of feedback from the panels that we were unable to act on because of federal requirements, resource limitations, and/or scope.

**Table 2:** Example protocol changes made in response to stakeholder feedback during project start-up

Stakeholder panel proposing change	Recommendation	How protocol changed
Systems	During interviews, ask about cultural preferences, benefits of receiving meals, and satisfaction	Updated our semi-structured interview guide to include questions on these topics
Systems	Include participants who are not English speaking	Translated all of our materials into Spanish; hired Spanish speaking staff
Systems	Expand the outcome measures proposed to include ER visits, avoidable hospitalizations, and primary care visits, which are commonly used by other health systems partners	Added new variables as exploratory outcomes to the analysis plan (i.e., ER visits, all-cause hospitalizations, potentially preventable hospitalizations)
Systems	Measure and/or report on variables of interest such as the reasons for missed deliveries, the characteristics of drivers, and the reasons participants end meals prematurely	Requested additional data on missed meals and the characteristics of drivers (paid/volunteer, length of tenure, etc.) from the participating programs; committed to reporting on the reasons why the participants ended their meal services prematurely
Systems	Include contextual data about the recruitment sites to address the generalizability of the study’s results	Added a series of in-person site visits and created a new question guide on organizational characteristics that is administered during site visits; developed summaries for each site to document variation in how older adults learn about each program, enrollment procedures, and meal delivery practices
Systems	Add more information about the referral process to problem solve recruitment issues	Held a meeting with the program partners to discuss the referral process in more detail; revised our Program Guide to clarify the recruitment steps for the sites; hosted a staff listening session as a site visit activity
Systems	Emphasize to potential research participants that their local programs are partners in the research study to improve recruitment rates	Updated our recruitment packet to display the logos of our research partners, included contact information for the local programs, and included letter of support from programs
Lived experience	Edit Welcome Letter to emphasize cash incentive that the study period lasts for 6 months, meals are free during the study, how spot on the waiting list is affected by participating in the study vs not	Updated the recruitment Welcome Packet emphasizing this information
Lived experience	Change confusing terminology in the consent form about “medical records”	Developed scripts for survey team to answer questions and included an FAQ in the Welcome Packet about use of participant records and data; worked with IRB and demonstrated the need to remove required language about “medical records”
Lived experience	Provide the weblinks to official project websites and partners so that participants can obtain more details about the study	Updated the recruitment Welcome Packet with contact information and websites for the study and participating programs
Lived experience	Educate participants that are randomly assigned to frozen meals about the steps in meal preparation	Developed scripts to prepare participants about the size of the frozen meals, freezer storage needs, and delivery times
Lived experience	During interviews, ask participants about their freezer space, other types of help they get from drivers	Updated the semi-structured interview guide to include questions on these topics

**Table 3:** Example suggestions for protocol changes made by stakeholders not enacted

Stakeholder panel proposing change	Recommendation	Challenge/barrier
Systems	Expand analysis to look at the cost savings/cost avoidance for participants and caregivers	Out of scope: Request is outside of our approved protocol and the expertise of our research team
Systems	Expand analysis to look at the use of subsidized housing	Out of scope: There are no data collection activities planned that could allow us to examine how outcomes differ by housing type
Systems	Employ new staff to assist programs with the administrative burden of the referral process	Budget limitation: Currently providing remuneration for each enrolled participant to participating sites, but unable to afford a dedicated staff member at each program to assist with referrals
Systems	Conduct in-person participant interviews to better assess the participant’s environment and contextual needs	Budget limitation: To implement would require resources for staff to travel to each participating site
Lived experience	Use in-person recruitment rather than a mailing as the first participant contact about the study	Budget limitation/out of scope: We did not budget for in-person recruitment visits and this would change the study design
Lived experience	Change the participation incentives to prepaid debit cards	University policy: We explored the possibility of offering debit cards in lieu of gift cards, but current university policy prohibited cash transfers without obtaining tax information
Lived experience	Replace the plain white mailing envelope with decorated, colorful envelope to get more attention	Budget limitation: Envelopes were pre-printed with logos; did not budget for designing and purchasing color envelopes

### Site visits

Given the pragmatic nature of this study, all our partner programs are instructed to provide services “as usual” to participants who are randomized to receive daily-delivered meals. Accordingly, we expect that services will—and should—vary depending on individual programs’ resources, administrative policies, and expectations of drivers as well as the needs of individual participants. However, we recognize that these variations may influence the overall quality of meal services provided, including the quality of social interactions and wellness checks, and warrant the need to track each program’s variations and evaluate their influence on service quality. To do this, the project director, with assistance from members of the research team, will complete site visits with all partner programs and gather data sources that capture variations in service delivery. These data sources include (a) observational data from driver ride-alongs, (b) journey mapping exercises, (c) focus groups and interviews with staff members, and (d) documents (e.g., menus) related to service delivery. Data from the site visits will be analyzed through open coding to identify key service variations that may influence daily-delivered meal service quality.

To evaluate service quality, we will leverage the knowledge and experiences of our advisory panels who will assign quality scores (1 = low quality, 2 = moderate quality, 3 = high quality) to each of the identified key service variations. Based on these rankings, our research team will apply adapted site-level methodologies [51, 52] to calculate a composite quality score for each site and conduct exploratory analyses to determine associations between program service quality and participant outcomes.

### Dissemination of study findings

Study results will be shared with multiple audiences. Prior to publication, results will be shared with our advisory panels and participating programs for their reactions, interpretations, and assistance in contextualizing findings. Members of the two advisory panels will work with the research team to create consumer-friendly summaries of findings to share with diverse audiences, including older adults receiving home-delivered meals and those participating in this study. Results, including any null results, will be submitted for publication to scientific, peer-reviewed journals.

## Discussion

The Deliver-EE Trial, greatly informed by stakeholder input, is the first fully powered randomized clinical trial to prospectively evaluate the comparative effectiveness of the two predominant meal delivery options that are contracted by healthcare entities. The knowledge generated from this randomized clinical trial will be of value to healthcare entities, community-based organizations, older adults, and their families, because it will identify the mode of meal delivery that best meets older adults’ needs and promotes community independence.

The proposed research will address key knowledge gaps identified by a multitude of stakeholders. Two recent reports by the National Academies of Science, Engineering, and Medicine called for rigorous research to understand effective and efficient ways for the healthcare sector to reduce food insecurity and other health-related social needs. Our proposed research addresses the need to identify “how interventions affect health, for which patients, via what mechanisms, and in what contexts,” put forward by the committee, and works toward their recommended goal to “Fund, Conduct, and Translate Research and Evaluation on the Effectiveness and Implementation of Social Care Practices in Health Care Settings” [14, 15].

In addition, this work meets the needs of other stakeholders, including payers, programs, and older adults. For example, interviews conducted with Medicare Advantage plan representatives who were considering offering benefits to address members’ social needs indicated that evidence about what services work and for whom was critical to their decision-making [53]. Relatedly, research conducted with Medicaid managed care organizations suggests that the ability to document effectiveness and cost savings is necessary for establishing partnerships and ensuring sustainability [54]. With increased demand for meal services and increasing costs associated with providing meals, home-delivered meal programs are seeking evidence about which mode of meal delivery most positively impacts their clients, recognizing that older adults differ in their preferences. Meals on Wheels program leaders and volunteer drivers speak to the importance of having this information to ensure the best service delivery for their clients. Finally, older adult stakeholders whom we have engaged in our project want to better understand what services allow them to remain at home, which is the most important outcome to them. Thus, this research is significant because it will provide evidence to guide stakeholders’ decisions and ultimately improve older adults’ health outcomes and quality of life.

As this study sought to incorporate stakeholders’ perspectives in the design and conduct of the trial, we identified limitations in our ability to be optimally responsive to stakeholders’ recommendations. Specifically, we experienced tensions between stakeholders’ suggestions and federal requirements, resource limitations, human subjects research approvals, and balancing recommendations that are out of scope of the current project. These limitations challenge the ability to have “authentic” engagement and be completely responsive to stakeholders’ input into research studies. We would encourage other researchers to report recommendations from their stakeholders that were not actionable to inform future study designs and improve engagement in research.

This pragmatic study is limited in its ability to have a truly pragmatic approach. Our study was designed to compare the effectiveness of two common approaches for delivering meals in a “real-world setting.” This approach was selected to increase external generalizability and provide an understanding of how these different modes of meal delivery impact older adults’ everyday experiences and health-related outcomes. However, according to the PRECIS-2 framework designed to assess pragmatism [55], some aspects of our study are limited in their pragmatism that must be acknowledged. Specifically, there were two domains in which we achieved limited pragmatism. First, our recruitment procedures are specific to this study. While we are recruiting participants from established waiting lists, the outreach and enrollment processes are not used in “usual care.” In addition, our follow-up procedures are less pragmatic in that the research team is conducting follow-up surveys to measure our secondary outcomes. Though home-delivered meals programs do regularly assess clients for eligibility, the survey questions and procedures that we use are not common practice. While patient-reported outcomes challenge the pragmatic nature of the trial, they were important to collect given the importance of these measures to stakeholders and their lack of availability in other datasets. These two limitations must be taken into consideration when considering the external validity and generalizability of findings that come from this trial to other settings and populations.

Another potential limitation to this study is in program implementation of the daily-delivered meals intervention. In keeping with the pragmatic spirit of this trial, we do not give specific instructions to programs about how to deliver meals, socialize with participants, or conduct wellness checks. Rather, programs are instructed to provide services that are consistent with their “usual” meal delivery practices—all of which are characterized as providing nutritious meals, promoting socialization, and promoting health and wellness. While older adults who receive the daily-delivered meals model report high satisfaction in national surveys [56], the human service model does lead to variation in how the meals, socialization, and wellness checks are delivered within and across programs. We are tracking this variation during the trial through site visits, participant interviews, and monthly program meetings; however, there are elements of the tailored socialization and wellness check that we are unable to measure for encounter with every participant.

In summary, we expect results from this pragmatic trial, informed by diverse panels of stakeholders representing both systems-level and lived experience perspectives, to produce evidence that is actionable, generalizable, and directly responsive to the needs of older adults and the programs that serve them.

## Trial status

Advarra Protocol #Pro00060759 (Version 3, dated November 30, 2023); recruitment start date: May 1, 2022; estimated primary completion date: December 31, 2024.

## Supplementary Information

Additional file 1. Appendix: Data Safety and Client Confidentiality Procedures.

## Data Availability

Study materials can be requested from the corresponding author.

## Abbreviations

Deliver-EE: Evaluating Effects of Meal Delivery on the Ability of Homebound Older Adults to Remain in the Community Via A Pragmatic, Two-Arm, Randomized Comparative Effectiveness Trial
CMS: Centers for Medicare and Medicaid Services
MOWA: Meals on Wheels America
IRB: Institutional Review Board
UCLA: University of California, Los Angeles
CDC: Center for Disease Control
DST: Dietary Screening Tool
ICC: Intraclass correlation coefficient
MAR: Missing at random
PRECIS: Pragmatic Explanatory Continuum Indicator Summary
AHRQ: Agency for Healthcare Research and Quality
PCORI: Patient-Centered Outcomes Research Institute
ER: Emergency room
FAQ: Frequently Asked Questions

## References

[1] Tucher EL, Keeney T, Cohen AJ, Thomas KS. Conceptualizing food insecurity among older adults: development of a summary indicator in the National Health and Aging Trends study. J Gerontol B Psychol Sci Soc Sci. 2021;76(10):2063–72. 33001172 10.1093/geronb/gbaa147 PMC8599055

[2] Ziliak J, Gundersen C. The state of senior hunger in 2018. 2020. Available from: https://www.feedingamerica.org/sites/default/files/2020-05/2020-The%20State%20of%20Senior%20Hunger%20in%202018.pdf. Cited 2024 Mar 27.

[3] Vaccaro JA, Huffman FG. Sex and race/ethnic disparities in food security and chronic diseases in U.S. older adults. Gerontol Geriatr Med. 2017;30(3):2333721417718344. 10.1177/2333721417718344 PMC5502940 28717673

[4] Murthy V. Food insecurity: a public health issue. Public Health Rep. 2016;131(5):655–7. 28123203 10.1177/0033354916664154 PMC5230819

[5] Wolfe WS, Frongillo EA, Valois P. Understanding the experience of food insecurity by elders suggests ways to improve its measurement. J Nutr. 2003;133(9):2762–9. 12949362 10.1093/jn/133.9.2762

[6] Gundersen C, Ziliak JP. Food insecurity and health outcomes. Health Aff (Millwood). 2015;34(11):1830–9. 26526240 10.1377/hlthaff.2015.0645

[7] Ziliak J, Gundersen C. The health consequences of senior hunger in the United States: evidence from the 1999-2016 NHANES. 2021. Available from: https://www.feedingamerica.org/sites/default/files/2021-08/2021%20-%20Health%20Consequences%20of%20Senior%20Hunger%201999-2016.pdf. Cited 2024 Mar 27.

[8] Berkowitz SA, Basu S, Meigs JB, Seligman HK. Food insecurity and health care expenditures in the United States, 2011–2013. Health Serv Res. 2018;53(3):1600–20. 28608473 10.1111/1475-6773.12730 PMC5980147

[9] Garcia SP, Haddix A, Barnett K. Incremental health care costs associated with food insecurity and chronic conditions among older adults. Prev Chronic Dis. 2018;15. Available from: https://www.cdc.gov/pcd/issues/2018/18_0058.htm. Cited 2024 Mar 27. 10.5888/pcd15.180058 PMC6130288 30171678

[10] AHIP. Access to healthy foods: social determinants of health. 2018 May. Available from: https://www.ahip.org/resources/access-to-healthy-foods-social-determinants-of-health. Cited 2024 Mar 27.

[11] Solomon LS, Kanter MH. Health care steps up to social determinants of health: current context. Perm J. 2018;22(22):18–139.

[12] Ellwood M, Downer S, Leib E, Greenwald R, Farthing-Nichol D, Luk E, et al. food is medicine opportunities in public and private health care for supporting nutritional counseling and medically-tailored, home-delivered meals. Harvard Library; 2014. Available from: http://nrs.harvard.edu/urn-3:HUL.InstRepos:32151131. Cited 2024 Mar 27.

[13] Willink A, DuGoff EH. Integrating medical and nonmedical services - the promise and pitfalls of the CHRONIC Care Act. N Engl J Med. 2018;378(23):2153–5. 29874539 10.1056/NEJMp1803292

[14] Martinez RM, Alper J, editors. Investing in interventions that address non-medical, health-related social needs: proceedings of a workshop. Washington, D.C.: National Academies Press; 2019. Available from: https://www.nap.edu/catalog/25544. Cited 2024 Mar 27. 31693330

[15] National Academies of Sciences, Engineering, and Medicine; Health and Medicine Division; Board on Health Care Services; Committee on Integrating Social Needs Care into the Delivery of Health Care to Improve the Nation’s Health. Integrating social care into the delivery of health care: moving upstream to improve the nation’s health. Washington (DC): National Academies Press (US); 2019. Available from: http://www.ncbi.nlm.nih.gov/books/NBK552597/. Cited 2024 Mar 27.

[16] Skira MM, Wang S, Konetzka RT. Trends in Medicaid home and community-based services waivers for older adults. Health Aff (Millwood). 2022Aug;41(8):1176–81. 35914198 10.1377/hlthaff.2022.00149 PMC12009609

[17] Murphy-Barron C, Buzby EA, Pittinger S. Overview of Medicare Advantage supplemental healthcare benefits and review of Contract Year 2022 offerings. Milliman Brief. 2022. Available from: https://bettermedicarealliance.org/wp-content/uploads/2022/03/MA-Supplemental-Benefits-Milliman-Brief_20220225.pdf.

[18] ACL. Nutrition Services | ACL Administration for Community Living. United States: Administration for Community Living; 2024 Feb. Available from: http://acl.gov/programs/health-wellness/nutrition-services. Cited 2024 Mar 27.

[19] Thomas KS, Gadbois EA, Shield RR, Akobundu U, Morris AM, Dosa DM. “It’s not just a simple meal. it’s so much more”: interactions between meals on wheels clients and drivers. J Appl Gerontol. 2020;39(2):151–8. 30569811 10.1177/0733464818820226 PMC7001144

[20] Berkowitz SA, Terranova J, Hill C, Ajayi T, Linsky T, Tishler LW, et al. Meal delivery programs reduce the use of costly health care in dually eligible medicare and medicaid beneficiaries. Health Aff (Millwood). 2018;37(4):535–42. 29608345 10.1377/hlthaff.2017.0999 PMC6324546

[21] Campbell AD, Godfryd A, Buys DR, Locher JL. Does participation in home-delivered meals programs improve outcomes for older adults? Results of a systematic review. J Nutr Gerontol Geriatr. 2015;34(2):124–67. 26106985 10.1080/21551197.2015.1038463 PMC4480596

[22] Shan M, Gutman R, Dosa D, Gozalo PL, Ogarek JA, Kler S, et al. A new data resource to examinE MEALS ON WHEels clients’ health care utilization and costs. Med Care. 2019;57(3):e15–21. 30001250 10.1097/MLR.0000000000000951 PMC6349554

[23] Thomas KS, Akobundu U, Dosa D. More than a meal? A randomized control trial comparing the effects of home-delivered meals programs on participants’ feelings of loneliness. J Gerontol B Psychol Sci Soc Sci. 2016;71(6):1049–58. 26613620 10.1093/geronb/gbv111

[24] Thomas KS, Mor V. Providing more home-delivered meals is one way to keep older adults with low care needs out of nursing homes. Health Aff (Millwood). 2013;32(10):1796–802. 24101071 10.1377/hlthaff.2013.0390 PMC4001076

[25] Thomas KS, Parikh RB, Zullo AR, Dosa D. Home-delivered meals and risk of self-reported falls: results from a randomized trial. J Appl Gerontol. 2018;37(1):41–57. 27798291 10.1177/0733464816675421 PMC6620777

[26] Institute of Medicine (US) Standing Committee on the Scientific Evaluation of Dietary Reference Intakes and its Panel on Folate, Other B Vitamins, and Choline. Dietary reference intakes for thiamin, riboflavin, niacin, vitamin B6, folate, vitamin B12, pantothenic acid, biotin, and choline. Washington (DC): National Academies Press (US); 1998. (The National Academies Collection: Reports funded by National Institutes of Health). Available from: http://www.ncbi.nlm.nih.gov/books/NBK114310/. Cited 2024 Mar 27. 23193625

[27] Chan AW, Tetzlaff JM, Gøtzsche PC, Altman DG, Mann H, Berlin JA, et al. SPIRIT 2013 explanation and elaboration: guidance for protocols of clinical trials. BMJ. 2013;8(346):e7586. 10.1136/bmj.e7586 PMC3541470 23303884

[28] USDA. U.S. Household Food Security Survey Module: Six-Item Short Form Economic Research Service, USDA. 2012 Sep. Available from: https://www.ers.usda.gov/media/8282/short2012.pdf.

[29] Hughes ME, Waite LJ, Hawkley LC, Cacioppo JT. A short scale for measuring loneliness in large surveys. Res Aging. 2004;26(6):655–72. 18504506 10.1177/0164027504268574 PMC2394670

[30] CDC. Health-Related Quality of Life (HRQOL) | CDC. 2022. Available from: https://archive.cdc.gov/www_cdc_gov/hrqol/index.htm. Cited 2024 Mar 27.

[31] Bailey RL, Mitchell DC, Miller CK, Still CD, Jensen GL, Tucker KL, et al. A dietary screening questionnaire identifies dietary patterns in older adults. J Nutr. 2007;137(2):421–6. 17237321 10.1093/jn/137.2.421

[32] Shan M, Thomas KS, Gutman R. A multiple imputation procedure for record linkage and causal inference to estimate the effects of home-delivered meals. Ann Appl Stat. 2021;15(1):412–36. 35755005 10.1214/20-aoas1397 PMC9222523

[33] Rutterford C, Copas A, Eldridge S. Methods for sample size determination in cluster randomized trials. Int J Epidemiol. 2015;44(3):1051–67. 26174515 10.1093/ije/dyv113 PMC4521133

[34] Hochberg Y, Tamhane AC. Multiple comparison procedures. USA: John Wiley & Sons, Inc.; 1987. p. 450.

[35] Dramé M, Lang PO, Jolly D, Narbey D, Mahmoudi R, Lanièce I, et al. Nursing home admission in elderly subjects with dementia: predictive factors and future challenges. J Am Med Dir Assoc. 2012;13(1):83.e17–20. 21493163 10.1016/j.jamda.2011.03.002

[36] Cepoiu-Martin M, Tam-Tham H, Patten S, Maxwell CJ, Hogan DB. Predictors of long-term care placement in persons with dementia: a systematic review and meta-analysis. Int J Geriatr Psychiatry. 2016;31(11):1151–71. 27045271 10.1002/gps.4449

[37] Pocock S. Clinical Trials: A Practical Approach. John Wiley & Sons; 2013. Available from: https://onlinelibrary.wiley.com/doi/book/10.1002/9781118793916.

[38] Imbens G, Rubin D. Bayesian inference for causal effects in randomized experiments with noncompliance. Ann Stat. 1997;25(1):305–27.

[39] Imbens GW, Rubin DB. Causal inference for statistics, social, and biomedical sciences: an introduction. Cambridge: Cambridge University Press; 2015. Available from: https://www.cambridge.org/core/books/causal-inference-for-statistics-social-and-biomedical-sciences/71126BE90C58F1A431FE9B2DD07938AB. Cited 2024 Mar 27.

[40] Frangakis CE, Rubin DB. Principal stratification in causal inference. Biometrics. 2002;58(1):21–9. 11890317 10.1111/j.0006-341x.2002.00021.x PMC4137767

[41] Hirano K, Imbens GW, Rubin DB, Zhou XH. Assessing the effect of an influenza vaccine in an encouragement design. Biostatistics. 2000;1(1):69–88. 12933526 10.1093/biostatistics/1.1.69

[42] Rubin DB. Causal inference through potential outcomes and principal stratification: application to studies with “censoring” due to death. Statist Sci. 2006;21(3). Available from: http://arxiv.org/abs/math/0612783. Cited 2024 Mar 27.

[43] Rubin D. Multiple imputation for nonresponse in surveys. New York: John Wiley & Sons, Ltd; 1987. Available from: https://onlinelibrary.wiley.com/doi/abs/10.1002/9780470316696.fmatter. Cited 2024 Mar 27.

[44] Strauss A, Corbin J. Grounded Theory Methodology. In: Handbook of Qualitative Research. Thousand Oaks, CA: Sage Publications; 1994. Available from: https://www.depts.ttu.edu/education/our-people/Faculty/additional_pages/duemer/epsy_5382_class_materials/Grounded-theory-methodology.pdf. Cited 2024 Mar 27.

[45] Ritchie J, Lewis J, Nicholls CM, Ormston R, editors. Qualitative research practice: a guide for social science students and researchers. Second edition. Los Angeles, Calif.: SAGE Publications Ltd; 2013. 456 p

[46] Creswell JW, Miller DL. Determining validity in qualitative inquiry. Theory Into Practice. 2000;39(3):124–30.

[47] Harrison J, Frampton S. Patient and family engagement in research in Era 3. J Am Coll Radiol. 2016 Dec; 13(12 Pt B):1622–4. 10.1016/j.jacr.2016.09.009 27888951

[48] Harrison J, Frampton S. Resident-centered care in 10 U.S. nursing homes: residents’ perspectives. J Nurs Scholarsh. 2017;49(1):6–14. 27676137 10.1111/jnu.12247

[49] Neuman P, Jacobson GA. Medicare advantage checkup. N Engl J Med. 2018;379(22):2163–72. 30428276 10.1056/NEJMhpr1804089

[50] Dobosenski A, Giglierano V. An Early Look at 2021 Medicare Advantage Benefits: Part I | Publications | Insights | Faegre Drinker Biddle and Reath LLP. Faegre Drinker; 2020. Available from: https://www.faegredrinker.com/en/insights/publications/2020/10/an-early-look-at-2021-medicare-advantage-benefits-part-i. Cited 2024 Mar 27.

[51] Harrison J, Balkan E, Gadbois E, Thomas K. A protocol for stakeholder engagement in Deliver-EE: a pragmatic randomized comparative effectiveness trial evaluating effects of meal delivery on the ability of homebound older adults to remain in the community. Contemporary Clinical Trials. In Press; 10.1016/j.cct.2024.107535 38614446

[52] Gaber J, Datta J, Clark R, Lamarche L, Parascandalo F, Di Pelino S, et al. Understanding how context and culture in six communities can shape implementation of a complex intervention: a comparative case study. BMC Health Serv Res. 2022;22(1):221. 35177040 10.1186/s12913-022-07615-0 PMC8855589

[53] Olson MB, McCreedy EM, Baier RR, Shield RR, Zediker EE, Uth R, et al. Measuring implementation fidelity in a cluster-randomized pragmatic trial: development and use of a quantitative multi-component approach. Trials. 2022;23(1):43. 35033176 10.1186/s13063-022-06002-8 PMC8761354

[54] Thomas KS, Durfey SNM, Gadbois EA, Meyers DJ, Brazier JF, McCreedy EM, et al. Perspectives of Medicare Advantage plan representatives on addressing social determinants of health in response to the CHRONIC Care Act. JAMA Netw Open. 2019;2(7):e196923. 31298711 10.1001/jamanetworkopen.2019.6923 PMC6628593

[55] Center for Health Care Strategies. Using Medicaid levers to support health care partnerships with community-based organizations. 2017. Available from: https://www.chcs.org/resource/using-medicaid-levers-support-health-care-partnerships-community-based-organizations/. Cited 2024 Mar 27.

[56] PRECIS-2. How to use PRECIS-2. University of Dundee; 2016. Available from: https://www.precis-2.org/Help/Documentation/HowTo. Cited 2024 Mar 27.

[57] AGID. 16th National Survey of Older Americans Act Participants (NSOAAP) Telephone Survey English Version. United States; 2022. Available from: https://agid.acl.gov/docs/16th_NSOAAP_2022_Instrument_11_14_2022-Final.pdf. Cited 2024 Mar 28.

[58] AHRQ. AHRQ quality indicators—guide to prevention quality indicators: hospital admission for ambulatory care sensitive conditions. Rockville, MD: Agency for Healthcare Research and Quality; 2001. Report No.: Pub. No. 02-R0203. Available from: https://www.ahrq.gov/downloads/pub/ahrqqi/pqiguide.pdf.

