# Supplementary material for: Evaluating effects of meal delivery on the ability of homebound older adults to remain in the community via a pragmatic, two-arm, randomized comparative effectiveness trial: study protocol for the Deliver-EE trial
Source: Trials. 2024 Nov 22;25:787. doi: 10.1186/s13063-024-08635-3 (PMC11583665; doi:10.1186/s13063-024-08635-3)
Supplement: Supplementary file 1 — Additional file 1. Appendix: Data Safety and Client Confidentiality Procedures. [file 13063_2024_8635_MOESM1_ESM.pdf]

Appendix: Data Safety and Client Confidentiality Procedures

Identifiable information will not be attached to any research documents, and all data will be identifiable only using the unique identifier specific to this study. All members of the research team have experience collecting data from participants and using secondary data that contains private health information. None of the study team members have had a breach of confidentiality associated with their research.

The risk of breach of confidentiality is through the protections set forth by the Center for Gerontology and Healthcare Research and the strict terms of our CMS Data Use Agreement (DUA). All participant data, including data obtained from CMS, will be stored on secure Center servers. At the conclusion of this study, or by the date of retention identified in the DUA, a CMS "Certificate of Disposition" certifying the proper destruction of all data obtained from CMS will be sent to CMS. Results of this research will be presented for large groups so that individuals cannot be identified. We will suppress the reporting of any data where the number of individuals is less than 11.

The Center for Gerontology and Healthcare Research maintains a high level of security and compartmentalization between projects that use different data sources. The Center's clustered computer systems are accessible only to authorized users, and only individuals working on this project will be authorized to access these data. Furthermore, Center employees have signed an oath of confidentiality, and its violation is sufficient grounds for immediate termination. Results of this research will be aggregated so that individuals are not identifiable. We will not be using a local storage device or removable media for data storage. More specifically, all data are received and stored securely on a central server, accessible in its raw form only to assigned custodians identified on each DUA, which includes all systems management staff. No data are ever exposed using any Internet protocol, and internally are secured by OpenVMS ACLs tied to both user identity and currently assigned duties. Original data materials are maintained securely by the receiving custodian, and returned to the providing authority as soon as practical. Data are backed up and secured against loss along with all other core assets on the OpenVMS and Windows server systems, and access to all backups is physically limited to systems management staff. System startup, shutdown, and configuration for all servers with access to secured data are likewise limited to internal systems management staff. Data custodians strip all external person-level identifiers, assign unique internal IDs to persons with the core data files, and make available individual-level and aggregate files to assigned project-specific staff within the Center, according to the authorized access provided to that project. All such provisioning is likewise done without use of any Internet-accessible protocols. Remote access to the Center's computing systems is protected by standard Discretionary Access controls, packet filtering, and Virtual Private Network protocols. Intentional violation of any of these protocols is grounds for dismissal from employment at the Center. At the termination of the Data Use Agreement, on or before the date on which authorized access to the data expires, all distribution, work-space, and archival backup copies of data will be destroyed (written over or otherwise made unreadable). We will maintain a counter-signed statement confirming the destruction of the restricted files.

Appendix: Informed Consent

Sponsor / Study Title:

Patient-Centered Outcomes Research Institute / "Deliver-EE: Evaluating Effects of Meal Delivery on the Ability of Homebound Older Adults to Remain in the Community Via A Pragmatic, Two-Arm, Randomized Comparative Effectiveness Trial

Principal Investigators:
Kali S. Thomas, PhD, MA
Johns Hopkins School of Nursing

Kimberly P. Bernard, PhD, MA
Brown University School of Public Health

Main Telephone:
401-863-9230 (24-Hour)

Main Mailing Address:
Brown University School of Public Health
121 South Main Street,
Providence, RI, 02912

You are invited to take part in a research study. Your participation is voluntary.

PURPOSE: We would like to better understand the health and well-being that come from receiving meals delivered to your home. We would also like to compare the benefits of different kinds of meal delivery. For example, some meals are delivered at lunchtime by someone from your local Meals on Wheels affiliated program. Other meals may be sent through the mail. We are trying to understand how these two options impact the health, wellbeing, and satisfaction of older adults.

You are being asked to enroll in this study because your name appears on a waiting list for meal services at your local Meals on Wheels affiliated program. About 2,300 people will take part in this study.

PROCEDURES: If you enroll in the study, you will immediately start receiving meals. You will either receive meals through an in-person delivery driver, or frozen meals through the mail. The number of meals will be the same. The type of meal delivery you get will be decided randomly by a computer program (similar to a flip of a coin). Neither you nor the research team can choose the type of meals you will receive. After six months, your participation in the study will

be complete. At the end of the 6-month study period, you will continue to receive meals from your local Meals on Wheels affiliated program based on your eligibility and the ability of the program to provide meals to you. You may also request a new delivery plan after the study ends.

You can request to stop receiving meals at any time.

As part of the study, you will also be asked to take part in up to two telephone interviews. You may be asked to participate in a third interview. These interviews will last 20 - 45 minutes each. During these interviews, we will ask you questions about your health, well-being, satisfaction with meals, caregiver support, and experiences with meal delivery services. You may refuse any interview, at any time. You may also decide not to answer certain questions during the interview.

Information about your medical services will be obtained from the Medicare program. Information about your meal delivery services will come from your local Meals on Wheels affiliated program. The information we collect for this study will be linked. Then, your personal identifiers will be deleted from the final dataset. Therefore, your personal information will never be shared with anyone outside of the research team.

At the end of the research study, we will mail you a summary of the main research findings. This summary will not be specific to you. It will also not contain personal information.

AUTHORIZATION TO USE AND DISCLOSE PROTECTED HEALTH INFORMATION: If you decide to be in this study, the research team will have health data about you. Health data may include:

> Your social security number, date of birth, medical diagnoses, dates of medical services, and other related information.

The research team may share health data about you with members of the Advarra IRB (an Institutional Review Board that reviews this study). Once your health data has been shared with authorized users, it may no longer be protected by federal privacy law and could possibly be used or disclosed in ways other than those listed here.

Your health data will be used to conduct and oversee this research. We will answer research questions such as:

> Does health improve after receiving meals through a home-delivery program?

At any time, you may take back your permission for the research team to use your health data by writing to the principal investigator at the address listed on the first page of this form. If you do this, you will be withdrawn from the study. You will no longer receive meals as part of the

study. No new health data about you will be collected after we receive your written request to withdraw. However, the health data that we already collected may still be used as described in this form.

You will not be able to be in the study if you do not give the research team permission to use your health data.

TIME INVOLVED: The study will take 40 - 110 minutes of your time over a six-month period.

COMPENSATION:

You will immediately receive five meals a week for six months at no charge. You will also receive a gift card each time you do an interview. You will be paid following each completed survey:

- Interview #1 will be 20 – 30 minutes. You will receive a 25 gift card.
- Interview #2 will be 30 – 45 minutes. You will receive a $50 gift card.
- Interview #3 will be 20 – 30 minutes. You will receive a $50 gift card.

There will be no charge to you for your participation in this study.

RISKS: There are minimal risks to you if you choose to be in this study. You may not like the meals you receive. If this occurs, you can ask to stop receiving meals. You may become tired or uncomfortable while answering some of the interview questions. If this occurs, you may skip questions or end the interview, at any time. You will be audio recorded for this study. It is possible that your voice may be recognizable and your identity become known.

BENEFITS: If you choose to be in the study, you will immediately start receiving meals. The information you share will be used to help improve meal delivery services. As a result, services that you and others like you may receive in the future through your local Meals on Wheels affiliated program may be improved.

CONFIDENTIALITY: We will keep your information private and safe. We use a unique number to label your information. Your name and address will be kept separate from that unique number. Therefore, no one will know who gave what answers. We will store all of your information in a secure computer database in a secure facility. Our research team is trained in protecting confidential information. We also have security measures in place to ensure the integrity and confidentiality of all data.

We use call recording software to record telephone interviews. This allows us to do quality control. For some calls, the interviewer may also be using a notebook and a digital recorder for

note taking. We will keep this information confidential just like all the other data. We will destroy all notes and recordings after the study is over.

Your de-identified data will be added to our research database. This database will be kept for at least seven years. Copies of our de-identified research database will also be shared with the funder of this study. Other researchers may be able to access this database for future research purposes. This de-identified database will not include any of your health data.

A description of this clinical trial will be available on http://www.ClinicalTrials.gov, as required by U.S. Law. This Web site will not include information that can identify you. The Web site will only include a summary of the results. You can search this Web site at any time.

VOLUNTARY: You do not have to be in this study if you do not want to be. Even if you decide to be in this study, you can change your mind and stop at any time. You may choose to not participate, or you may withdraw from the study for any reason without penalty or loss of benefits to which you are otherwise entitled. If you are no longer in the study, you will stop receiving meals. You can also skip any interview questions or end an interview at any time.

The principal investigator or the sponsor can stop your participation at any time without your consent for the following reasons:

- If you fail to follow directions for participating in the study;
- If it is discovered that you do not meet the study requirements;
- If the study is canceled; or
- For administrative reasons.

This study is for research purposes only. Your only alternative is to not participate in this study. Any new important information that is discovered during the study and which may influence your willingness to continue participation in the study will be provided to you.

WHOM TO CONTACT ABOUT THIS STUDY: During the study, if you have questions, concerns or complaints about the study such as:

- Payment or compensation for being in the study;
- Your responsibilities as a research participant;
- Eligibility to participate in the study;
- The principal investigator's or study site's decision to exclude you from participation.

Please contact the principal investigator at the telephone number listed on the first page of this consent document.

An institutional review board (IRB) is an independent committee established to help protect the rights of research participants. If you have any questions about your rights as a research participant, contact:

- By **mail**:
  Study Subject Adviser
  Advarra IRB
  6100 Merriweather Dr., Suite 600
  Columbia, MD 21044
- Or call **toll free**: 877-992-4724
- Or **email**: adviser@advarra.com

Please reference the following number when contacting the Study Subject Adviser: Pro00060759.
